# Community-based Agency Delivery of Parent-Child Interaction Therapy: Comparing Outcomes for Children with and Without Autism Spectrum Disorder and/or Developmental Delays

**DOI:** 10.1007/s10803-022-05755-0

**Published:** 2022-11-02

**Authors:** Lauren B. Quetsch, Rebecca S Bradley, Laurie Theodorou, Kathleen Newton, Cheryl B. McNeil

**Affiliations:** 1 University of Arkansas, Fayetteville, Arkansas United States; 2 Oregon Health Authority, Salem, OR United States; 3 West Virginia University, Morgantown, WV United States; 4 University of Florida, Gainesville, FL United States

**Keywords:** autism spectrum disorder, developmental delays, Parent-Child Interaction Therapy, disruptive behaviors, community-based providers

## Abstract

While externalizing behaviors are common among children with autism spectrum disorder (ASD), there is a shortage of specialist community-based clinicians to provide treatment. Parent–Child Interaction Therapy (PCIT), an intervention designed to reduce child disruptive behaviors, may be effective for families of children with ASD but has rarely been studied outside of university-based research settings. We examined the effectiveness of PCIT delivered for children with (N = 109) and without (N = 2,324) ASD/developmental delays (DD) across community-based agencies in Oregon. Findings revealed significant reductions in disruptive behavior and positive changes in the parent-child relationship in both groups. These findings support PCIT as an efficacious intervention for children with ASD/DD and demonstrate PCIT’s promise in community-based agencies with non-specialized clinicians.

Autism spectrum disorder (ASD) is a neurodevelopmental condition characterized by deficits in social communication and reciprocity as well as stereotypic behaviors and interests (American Psychiatric Association, 2022). Families of young children with autism often face myriad co-occurring conditions. One of the most prevalent issues is child disruptive behaviors which occur in 12–48% of the population (see Hossain et al., 2020, for a review; Mazurek et al., 2013). These behaviors may include anger outbursts, irritability, aggression, noncompliance, and oppositionality (Burke et al., 2002). Autism researchers have long posited that problem behaviors in ASD function in the context of multiple factors including adaptive functioning/social communication deficits, reinforcement in the environment, and broader predisposing biological characteristics (Kanne & Mazurek, 2011; Smith et al., 2016). Although disruptive behavior in youth with ASD may clinically differ from typically developing youth (Beauchaine et al., 2010), research indicates that approximately one in four children with ASD meets criteria for a comorbid disruptive behavior disorder (Kaat & Lecavalier, 2013). The presence of these disruptive behaviors can predict higher levels of parental stress, reduce a child’s ability to benefit from early intervention services, and impede a child’s school functioning (Postorino et al., 2019; Soke et al., 2018). Furthermore, disruptive behaviors may also limit a child’s success in other ASD-specific treatment modalities meaning even if the child does have access to care, they may not benefit as much as their counterparts without such concerns (Jang et al., 2011).

## Clinical Need for ASD Services

Behavioral interventions focusing on the child, such as applied behavioral analysis (ABA), have demonstrated success in reducing disruptive behavior in youth with ASD (Doehring et al., 2014), but a shortage of specialty clinicians trained in ASD symptomology limits children’s access to treatment (Xu et al., 2019; Zhang & Cummings, 2020). Indeed, less than 40% of children with ASD will receive early behavioral intervention (Zablotsky et al., 2015). This service inaccessibility is especially prevalent given the threefold increase of ASD cases since 2000 (current prevalence is 1 in 44 children; CDC, 2000; 2021). Currently, nearly every state in the U.S. falls below the necessary provider-to-child ratio, with the highest rates of ABA service access clustered in wealthy, highly insured counties (Yingling et al., 2021; Zhang & Cummings, 2020). Given the lack of autism specialists, non-specialists are frequently faced with treating the comorbid conditions of children with ASD (e.g., disruptive behaviors; Naveed et al., 2019) with community-based agencies shouldering the bulk of these demands as they serve a wide range of needs for youth in their regions (Brookman-Frazee et al., 2012).

## Community-based Treatment Options

Regrettably, many community-based providers feel ill-prepared to tackle the unique needs of children with autism even with these children being referred to their clinics at high rates (Brookman-Frazee et al., 2012). Providers are often interested in utilizing evidence-based practices to address child disruptive behaviors; however, researchers conducting efficacy and effectiveness trials of treatment protocols have historically excluded children with ASD. This means that community providers are left with few evidence-based treatment options or skills needed to attend to the unique needs of children with ASD entering their clinics. Therefore, little is known about the effectiveness of treatment as usual or evidence-based treatment implementation in community settings when it comes to treating youth with ASD or with other developmental disorders (DD).

### Parent-Child Interaction Therapy

Fortunately, Parent-Child Interaction Therapy (PCIT; a parent-training program to promote positive parent-child relationships, reduce child aggression, and decrease noncompliance) does not require autism specialization yet has recently demonstrated success for ASD populations (McNeil & Hembree-Kigin, 2010; McNeil et al., 2019). PCIT is a behavioral parent training intervention with extensive empirical support in treating young children with disruptive behaviors (Thomas et al., 2017). Grounded in attachment and learning theories (Eyberg, 1988), PCIT is centered around parent-child play and comprised of two phases: Child-Directed Interaction (CDI) followed by Parent-Directed Interaction (PDI; Eyberg & Funderburk, 2011). CDI focuses on strengthening the caregiver-child relationship and improving positive parenting strategies. PDI increases child compliance and reduces child disruptive behaviors by teaching parents consistent discipline skills. PCIT’s live coaching component makes it distinctive from other parent training programs as parents receive immediate feedback while interacting with their child. A PCIT Certified Therapist delivers treatment via a one-way mirror to help parents attain therapeutic skills while playing directly with their child. PCIT follows gold-standard practices in training clinicians in evidence-based interventions (Frank et al., 2020); certified therapists receive an intensive training and continually participate in supervision and consultation with other PCIT practitioners.

There is a wealth of evidence supporting PCIT as an efficacious treatment for youth in the general population. In one recent meta-analysis, PCIT demonstrated effectiveness in reducing child disruptive behavior and improving the parent-child relationship with a large mean effect size (*d* = 1.65) across twelve studies (Ward et al., 2016). Moreover, recent research has also demonstrated PCIT’s promise in reducing disruptive behavior in youth with ASD (e.g., Parladé et al., 2019) and developmental delays (Bagner & Eyberg, 2007; Bagner et al., 2010). In a recent study by Zlomke & Jeter (2020), researchers found that PCIT significantly improved disruptive behaviors in both an ASD and a non-ASD group, such that diagnostic status (ASD vs. non-ASD) was not a significant predictor of disruptive behavior reductions. Similarly, PCIT was shown to be effective for youth with developmental delays in two formative randomized control trials (for a discussion, see Ros et al., 2016).

PCIT has been widely disseminated across thousands of sites in the United States including several statewide initiatives (Scudder et al., 2017). Given the wide availability of PCIT providers and the promising outcomes of non-adapted PCIT for youth with ASD, it stands to reason that PCIT may be a viable option for families of youth with ASD seeking help for child disruptive behaviors in community-based agencies.

Yet, PCIT has largely been studied in university-based research studies for this population (McNeil et al., 2019). More exploration is needed to see how standard PCIT performs in community-based clinics by (non-specialist) mental health providers untrained in research initiatives and who may deliver treatment with variable levels of fidelity (e.g., Lieneman et al., 2019). Moreover, few studies have explored comparisons between children with ASD and neurotypical youth on variables such as the amount of time in treatment and outcomes of child disruptive behaviors to determine if there are differences in treatment impact (e.g., McInnis et al., 2020). With evidence of PCIT’s efficacy among youth with ASD mounting, there is a need to continue to explore the effect of PCIT on families of children with ASD in a community-based treatment setting.

## Current Study

The current project analyzed an existing, community-based dataset comprised of children who received PCIT services across the state of Oregon. Oregon’s legislature began funding community implementation of PCIT in 2004, and currently, PCIT is provided in over 45 locations across the state (Oregon Health Authority, 2021). This widespread dissemination of PCIT has produced one of the largest samples in the history of PCIT research. Data for youth were tracked by community clinicians on diagnoses, caregiver information, length of time in treatment, number of PCIT sessions, outcomes for relationship enhancement and graduation, as well as intensity and number of disruptive behaviors. The research team hypothesized that children with ASD would have similar rates of treatment length, relationship enhancement, graduation, and reductions of disruptive behaviors when compared to children without an ASD diagnosis.

## Method

### Participants

The present study analyzed data from 2,435 child-caregiver dyads (regardless of diagnostic status) receiving PCIT services in community mental-health agencies across the state of Oregon. The present study represents one of the largest studies ever conducted with families receiving PCIT (e.g., Lieneman et al., 2019). Overall, 45 children were identified as having ASD and 70 were diagnosed with DDs (with some overlap). In the present study, diagnoses were identified from billing records. The state of Oregon does not allow for PCIT providers to reimburse for treatment for a diagnosis of ASD unless clinicians are also practicing ASD treatments approved under the Oregon Health Authority (e.g., ABA, medical therapy; Oregon Health Authority, 2021). This billing barrier yielded many children having additional diagnoses of autism or another unspecified DD (e.g., pervasive developmental disorder, unspecified disorder of psychological development, receptive/expressive language disorder) to indicate pervasive developmental conditions but allow for reimbursement of a billable code (e.g., conduct disturbance, adjustment disorder). However, the researchers did not include intellectual disability in the categorization of DD as this was a separate code (although not explicitly explored in the present analyses). Moreover, while mental health providers can indicate concerns with DD, many are not permitted to provide an ASD diagnosis which may have limited the number of children classified with ASD. When exploring demographic characteristics and child outcomes (e.g., intensity of disruptive behaviors) of both groups (i.e., ASD, DD), no significant differences arose between the classifications (*p*’s > 0.05); therefore, outcomes for the larger group were combined to avoid the possibility of missing any child with autism in the sample (*N* = 109).

Families were located in rural (*n* = 1,400, 57.5%), urban (*n* = 984 = 40.4%), and frontier (*n* = 50; 2.1%) agencies (missing: *n* = 1, 0.0%). Children were largely male (*n* = 1,535, 63.3%), averaged 5 years and 9 months of age, spoke English as a primary language (*n* = 1,988; 81.7%), and were mostly White (*n* = 1,112; 45.8%; next highest = Unknown [*n* = 1,013; 41.7%], Hispanic or Latinx [*n* = 181; 7.5%]). Caregivers were mostly female (*n* = 2,049; 84.2%), parents (*n* = 2,067; 84.9%), and were on Medicaid (*n* = 2,423; 99.5%). See Table 1.

**Table 1:** *Demographic Comparisons for Families of Children with and without Autism Spectrum Disorder and/or a Developmental Delay*

	Total			Comparison			ASD/DD			
	*N*	*M*		*N*	*M*(*SD*)		*N*	*M*(*SD*)	*t*	*p*
Child Age (Months)	2,433	60.75		2,324	60.80(18.15)		108	59.84(17.98)	0.53	0.59
	* N*	%		*N*	%		*N*	%	*χ* ^2^	*p*
Agency Location	2,433			2,324			109		2.13	*0.35*
Urban	984	40.4		947	40.7		37	33.9		
Rural	1,399	57.5		1,330	57.2		69	63.3		
Frontier	50	2.1		47	2.0		3	2.8		
Child Gender	2,424			2,315			109		14.89	< 0.001
Male	1,535	63.3		1,447	62.5		88	80.7		
Female	889	36.7		868	37.5		21	19.3		
Child Ethnicity/Race	2,427			2,318			109		28.20	0.11
White	1,112	45.8		1,055	45.5		57	52.3		
Hispanic/Latinx	181	7.5		174	7.5		7	6.4		
Black	43	1.8		38	1.6		5	4.6		
Pacific Islander/ Asian	11	0.5		10	0.4		1	0.9		
American Indian/ Alaskan Native	44	1.8		42	1.8		2	1.8		
Other	23	0.9		23	1.0		0	0		
Unknown	1,013	41.7		976	42.1		37	33.9		
Child Primary Language	2,434			2,325			109			
English	1,988	81.7		1,899	81.7		89	81.7	6.11	0.11
Spanish	193	7.9		179	7.7		14	12.8		
Other	253	10.4		247	10.6		6	5.5		
Caregiver Sex	2,434			2,325			109		0.05	0.98
Male	384	15.8		367	15.8		17	15.6		
Female	2,049	84.2		1,957	84.2		92	84.4		
Caregiver Role	2,434			2,325			109		3.14	0.93
Parent	2,067	84.9		1,972	84.8		95	87.2		
Grandparent	137	5.6		130	5.6		7	6.4		
Step Parent	45	1.8		44	1.9		1	0.9		
Aunt/Uncle	40	1.6		38	1.6		2	1.8		
Foster Parent	139	5.7		135	5.8		4	3.7		
Other	6	0.2		6	0.3		0	0		

*Notes.* ASD = autism spectrum disorder; DD = developmental delay; PCIT = Parent-Child Interaction Therapy. Comparison group reflects youth without ASD and/or DD. Demographic comparisons describe the entire sample of families

## Procedure

All families received standard PCIT. Clinicians were not provided with additional training on ASD/DD populations or given consultation on how to appropriately adapt the treatment model for this unique population. For the present study, the agency in which a family received PCIT collected participants’ data and treatment outcomes as part of routine reporting procedures. Importantly, while reporting these data were required by the involved agencies, clinicians were not trained in research or data collection procedures; therefore, data collection was limited in both breadth and depth, with many families having missing data. Data were retained in the state-affiliated agency’s database, deidentified, and retrieved following the approval of the IRBs from the research teams’ associated universities.

## Measures

### Demographics

Key demographic variables were collected by state-affiliated community mental health agencies for families including child sex, age, ethnicity/race, and primary language spoken as well as caregiver sex and relationship to the child receiving treatment. Mental health clinics were classified by the population they served as urban (< 10 miles from population centers of ≥ 40,000), rural (> 10 miles from population centers of ≥ 40,000), or frontier (counties with < 6 people per square mile) as defined by the Oregon Office of Rural Health (2020).

### Length of Treatment and Number of PCIT Sessions

The total length of treatment (in weeks) as well as number of PCIT sessions were recorded by the clinician providing services for each family. Parents are required to meet predetermined goal criteria for the targeted parenting skills before proceeding from the first to second phase of treatment (i.e., CDI to PDI). While the total number of PCIT sessions is frequently cited as lasting between 12 and 14 sessions (Chaffin et al., 2011), PCIT delivery in community-based agencies has often lasted longer (e.g., 17–20 sessions; Quetsch et al., 2020), indicating parents need more direct coaching sessions to meet goal criteria in these settings. Importantly, community implementation researchers have also noted that increased program length may be a barrier to treatment and contribute to attrition (Chen & Fortson, 2015). Length of treatment and number of PCIT sessions allow for comparisons of the treatment process in families of children with and without ASD/DD.

### Improvement and Graduation

Clinicians reported on their impressions of family improvement in treatment (1 = *No Improvement*, 2 = *Some Improvement*). To meet PCIT graduation criteria, families must (1) achieve goal criteria in both CDI and PDI phases, (2) report their child is no longer experiencing clinically significant disruptive behavior difficulties on the Eyberg Child Behavior Inventory Intensity subscale, and (3) report confidence in being able to handle their child’s behavior independently. PCIT clinicians determined whether family graduation status was met (*yes/no*).

### Eyberg Child Behavior Inventory

Child disruptive behavior was measured using the Eyberg Child Behavior Inventory (ECBI; Eyberg & Pincus, 1999). The ECBI is a widely used 36-item caregiver-report measure for the (a) frequency of child behaviors (Intensity scale; ratings: 1 = *Never* to 7 = *Always*) and (b) caregiver perception of concern for each behavior (Problem scale; ratings: *yes/no*). Previous studies have indicated adequate levels of reliability and validity (Eisenstadt et al., 1994; Eyberg & Pincus, 1999), and the ECBI has demonstrated good internal consistency in typically developing children (Cronbach’s alpha values: 0.93–0.95; Colvin et al., 1999). In a psychometric analysis of the ECBI in youth with ASD, Jeter and colleagues (2017) found that the ECBI had good to excellent reliability with Cronbach’s alpha values of 0.88 and 0.92 for the Problem and Intensity scales respectively. While ECBIs are delivered at every weekly session (McNeil & Hembree-Kigin, 2010), clinicians in the present study only recorded ECBIs at *pre-treatment* (prior to the start of PCIT) and at the last recorded PCIT session the family attended (referred to in this study as *post-treatment*). Change scores for each ECBI scale (i.e., Intensity, Problem) were calculated by subtracting the ECBI score from the last session the family attended from the pre-treatment score. Negative ECBI scores indicate a worsening of child behaviors, while positive scores indicate improvement in child behaviors over time. ECBI scores are required to be below clinical levels (i.e., 114) prior to graduation from treatment.

## Analysis Plan

Comparisons between demographic characteristics were performed using independent samples *t*-tests and chi-square analyses to determine if differences arose between families with or without a child with ASD/DD. To examine potential differences in treatment processes between groups, independent samples *t-*tests were conducted on the number of days participants were in treatment and the number of PCIT sessions attended. Therapist-reported relationship enhancement and rates of graduation were examined between groups using chi-square analyses.

Differences in pre- to post-treatment ECBI scores between groups were compared using a mixed analysis of variance. While our demographic comparisons and descriptive statistics reflect the entire sample, families with fewer than four sessions (*N* = 1,490) and/or with missing data (*N*_*Intensity*_ = 733, *N*_*Problem*_ = 745) were excluded from analyses of ECBI scores (some families fitting into both categories). A total of 804 families were included in EBCI Problem Scale analyses and 769 families were included in ECBI Intensity scale analyses. In previous work with this sample, four complete sessions were estimated to be the smallest possible dose of PCIT for meaningful behavior change and skill acquisition to occur (Lieneman et al., 2019). It was assumed that the first three PCIT sessions typically cover intake assessments and introduction to parent training (CDI teach). Following CDI teach, families receive parent coaching. It is also assumed that only families who received at least one coaching session may experience change from participating in PCIT; therefore, change would be observed at the fourth session. Preliminary analyses explored if pre-treatment differences were present for ECBI scores between families who attended less than four sessions versus those attended four sessions or more. Outcomes indicted no significant differences between groups on either the ECBI Problem or Intensity subscales (all *p*-values > 0.05).

All families were included in the present sample rather than using a matched sample comparison as it ensured full representation of families who received services within the Oregon agencies (rather than a select group) during this period. Due to limited demographic information, the research team was also concerned that matched samples still would not accurately represent samples that were similar. For example, given the large portion of participants with “unknown” racial identity, matching may have either (1) lumped this broad classification as one group therefore unintentionally creating a homogenization of racially or ethnically diverse families or (2) required that these individuals not be included in the matching process, again possibly unintentionally eliminating the experiences of racially or ethnically diverse families. Further, the intention of this project was to see how outcomes of individuals with autism or developmental delays in PCIT compared to all other families receiving PCIT (rather than just exploring the experiences of only a few families). Therefore, all eligible families were included in the analysis plan.

## Results

All outliers were removed prior to mean comparisons between groups as determined via Tukey’s method. The following variables were transformed due to non-normal distributions to perform *t*-test comparisons: length of treatment, number of PCIT sessions. All descriptive statistics (demographics, length of treatment, number of PCIT sessions, relationship enhancement, and graduation) describe the entire sample of families. As we assumed at least four PCIT sessions are needed to promote meaningful change, families with missing data and/or less than four PCIT sessions attended were excluded from ECBI scale score analyses.

## Demographics

Families of children with and without ASD/DD were compared on demographic variables (i.e., child age, sex, ethnicity/race, primary language; caregiver sex, relationship to child in treatment). The only significant difference that arose between demographic characteristics was child sex, *χ*^2^(1) = 14.89, *p* < .001. This finding indicates that there were proportionally more males (fewer females) receiving PCIT who were diagnosed with ASD/DD than children without ASD/DD (identified in the present study as the “comparison” group). All other demographic variables were not significantly different between groups (Table 1).

## Length of Treatment and Number of PCIT Sessions

Families in both groups were compared on the total number of days they were enrolled in PCIT as well as the number of treatment sessions they attended. No differences arose between families of children with ASD/DD as compared to the comparison group. Overall means indicated families were enrolled 127 days (approximately 4 months, 1 week) and attended between 9 and 11 sessions (Table 2). In the overall sample, the median days in treatment was 99, while the mode was 1. The median PCIT sessions attended was 7, while the mode was 2.

**Table 2:** *Comparisons of Length of Treatment and Number of Sessions in PCIT Between Groups*

	Total			Comparison			ASD/DD			
	*N*	*M*(*SD*)		*N*	*M*(*SD*)		*N*	*M*(*SD*)	*t*	*p*
Length of Treatment (Days)	2,044	126.64(114.19)		1,974	126.73(114.78)		70	124.06(96.93)	− 0.52	0.61
Number of PCIT Sessions	1,320	9.50(8.34)		1,271	9.41(8.22)		49	11.78(10.83)	-1.03	0.30

*Notes.* ASD = autism spectrum disorder; DD = developmental delay; PCIT = Parent-Child Interaction Therapy. Comparison group reflects youth without ASD and/or DD. Comparisons of treatment length and number of sessions include the entire sample of families

## Improvement and Graduation

Therapist-report of relationship enhancement and client graduation from treatment were compared between children with and without ASD/DD using chi-square analyses (Table 3). Outcomes indicated no significant differences in reported relationship enhancement between groups, *χ*^2^(1) = 0.16, *p* = .69. Similarly, graduation rates did not differ among families of children with and without ASD/DD, *χ*^2^(1) = 0.96, *p* = .91.

**Table 3:** *Comparisons of Relationship Enhancement and Graduation Rate Between Groups*

	Total			Comparison			ASD/DD			
	*N*	%		*N*	%		*N*	%	*χ* ^2^	*p*
Relationship Enhancement	2,035			1,966			69		0.16	0.69
Some Improvement	1,311	64.4		1,265	64.3		46	66.7		
No Improvement	724	35.6		701	35.7		23	33.3		
Graduation from Treatment	2,029			1,961			68		0.96	0.91
Yes	363	17.8		351	17.9		12	17.6		
No	1,666	82.1		1,610	82.1		56	82.4		

*Notes.* ASD = autism spectrum disorder; DD = developmental delay. Comparison group reflects youth without ASD and/or DD. Comparisons of relationship enhancement and graduation rate include the entire sample of families

## Child Disruptive Behavior

**Figure 1:**
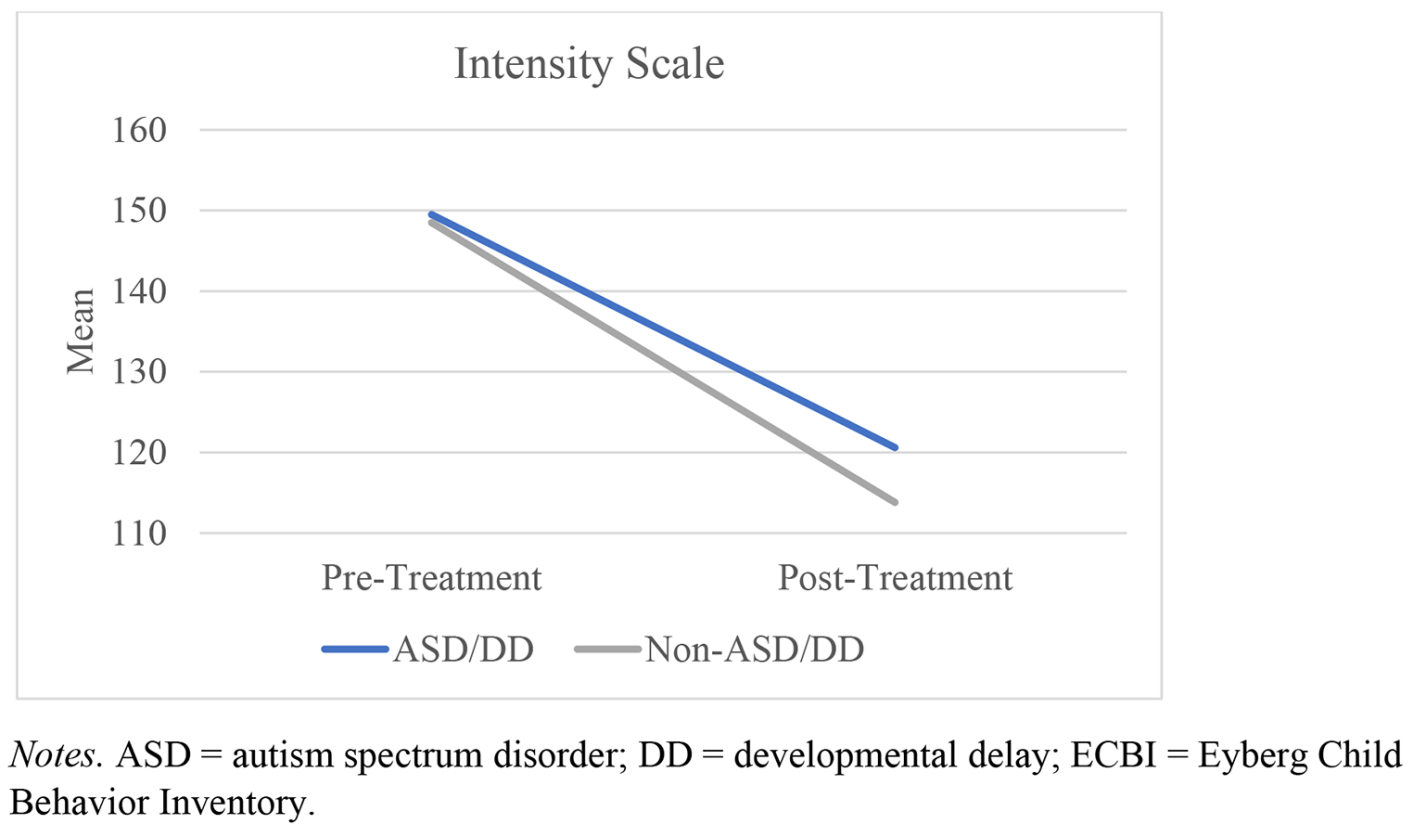
*Change in Mean Scores on the ECBI Intensity Scale Over Time*

**Figure 2:**
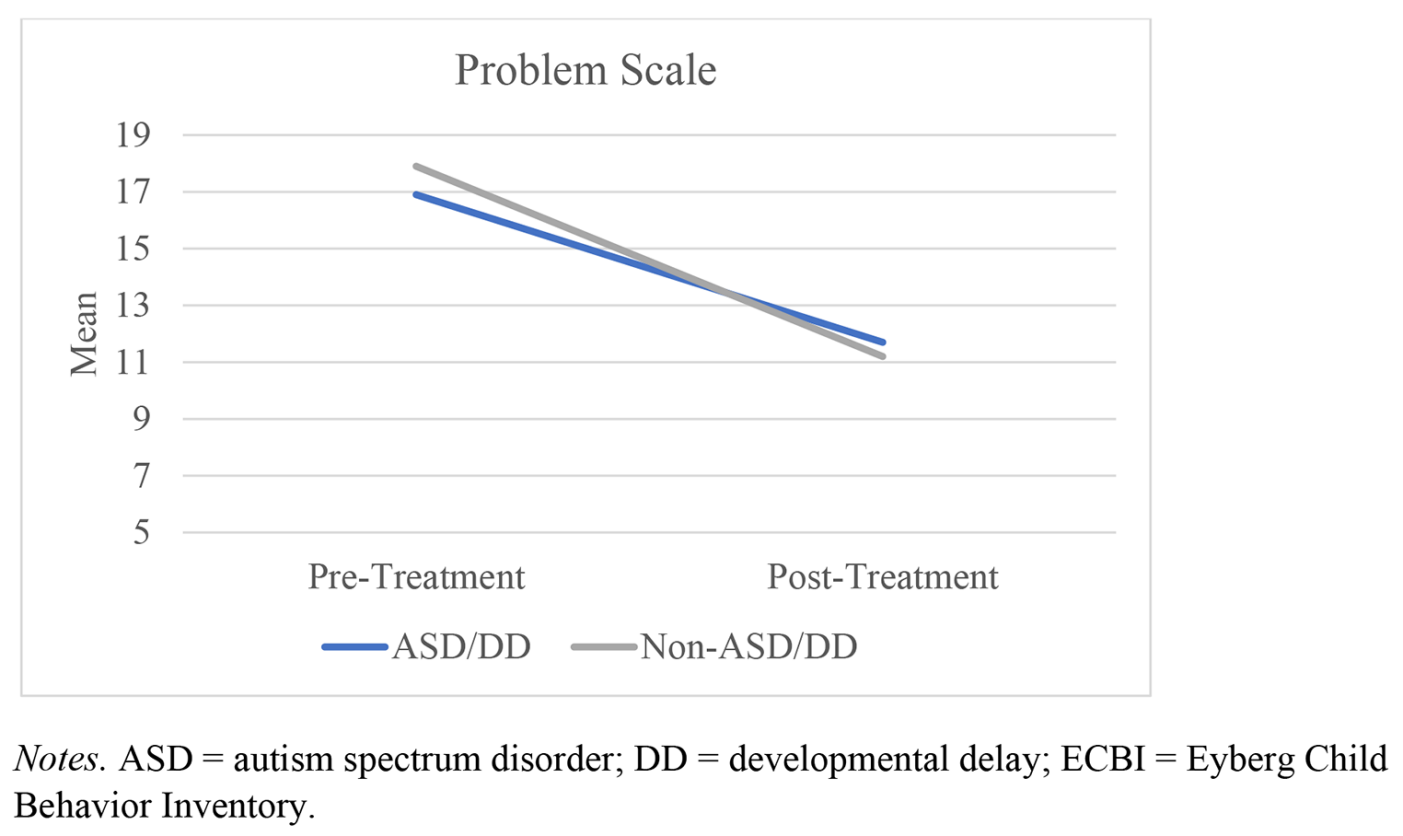
*Change in Mean Scores on the ECBI Problem Scale Over Time*

A mixed ANOVA was conducted to determine the effects of between-group (group: ASD/DD vs. comparison) as well as within-group (time: pre- and post-treatment) differences on measures of both ECBI Intensity and Problem scores (Table 4). Only families who attended at least four sessions of PCIT were included in the present analyses. All assumptions were met. Outcomes indicated that while there was a within-group effect for time, *F*(1,802) = 100.58, *p* < .001, η_p_^2^ = 0.11, there was no interaction effect (group X time), *F*(1,802) = 0.84, *p* = .36, η_p_^2^ = 0.001 (Fig. 1). For the Problem scale, outcomes yielded a within-group effect for time, *F*(1,794) = 58.51, *p* < .001, η_p_^2^ = 0.07, but there was no interaction effect (group X time), *F*(1,794) = 0.83, *p* = .36, η_p_^2^ = 0.001 (Fig. 2). These findings indicate that caregiver-report of the intensity of child disruptive behaviors and views of whether these behaviors were problematic both became significantly better after receiving at least four sessions of PCIT, but there were no significant differences between the two groups on child intensity or level of disruptive behaviors over time.

**Table 4:** *Mean Comparisons and Analysis of Variance of ECBI Intensity and Problem Scores For Each Group Over Time (Pre- to Post-Treatment)*

Measure		Pre-Treatment					Post-Treatment								
	Comparison			ASD/DD			Comparison			ASD/DD					
	*N*	*M*	*SD*	*N*	*SD*	*M*	*N*	*M*	*SD*	*N*	*M*	*SD*		*F*(1, 802)	η_p_^2^
**ECBI Intensity**	771	148.5	36.1	33	149.5	25.9	771	113.8	41.3	33	120.6	35.5		100.58***	0.11
														* F*(1, 794)	η_p_^2^
**ECBI Problem**	763	17.9	8.6	33	16.9	7.4	763	11.2	9.8	33	11.7	8.9		58.51***	0.07

*Notes.* ASD = autism spectrum disorder; DD = developmental delay; PCIT = Parent-Child Interaction Therapy. Comparison group reflects youth without ASD and/or DD. Families with missing data and/or less than four PCIT sessions were excluded from ECBI scores analyses

***p < .001

## Discussion

The current study examined PCIT delivered for children with and without ASD/DD across community-based clinics in Oregon. Findings revealed significantly more males receiving PCIT than females for children with ASD/DD as compared to children without ASD/DD. This proportion is expected as ASD is diagnosed more frequently in males than females (1 male per every 4 females; CDC, 2020). Outcomes also yielded significant reductions in parent-reported frequency of disruptive behaviors (ECBI Intensity scores) and parental perceptions of problematic child behaviors (ECBI Problem scores) among all children who received at least four sessions of PCIT. Additionally, no group differences were observed for length of time in treatment, number of sessions attended, therapist-reported relationship enhancement or graduation rates, or in Intensity or Problem scores from pre- to post-treatment. This study represents one of the first wide-scale explorations of the delivery of standard PCIT for children with ASD/DD by non-specialized clinicians at the community level (see also McInnis et al., 2020).

To determine similarities and differences in how long families were involved in PCIT, group comparisons were explored for the length of time spent in treatment as well as the number of sessions attended. No differences arose between families of children with ASD/DD and the comparison group. These outcomes indicate that treatment was not extended for families of children with ASD/DD which is reflective of several PCIT studies (see Owen et al., 2019 for a review), although increased number of sessions has been found in previous literature (Zlomke et al., 2017). Families of youth with and without ASD/DD also graduated from PCIT at similar rates. Given the additional challenges that can arise and adaptations that are frequently required when implementing PCIT with ASD/DD children (Owen et al., 2019), these findings are promising for community-based clinics. Of note, clinicians in the present study were not known to have extensive experience or expertise in ASD/DD. Additionally, dissimilar to other research exploring PCIT with ASD/DD populations, clinicians in this study were not part of a university-based research team thus demonstrating PCIT’s utility within real-world settings (McInnis et al., 2020).

Next, families with and without children with ASD/DD were compared on therapist-reported parent-child relationship enhancement. No differences were found indicating therapists perceived parents and their children with ASD/DD as demonstrating similar levels of relationship enhancement compared to the comparison group. Although the measure used in the present study was rudimentary, these findings are valuable for several reasons given that only a portion of the sample of youth was diagnosed with ASD. First, children with ASD are characterized as having deficits in the development, maintenance, and comprehension of relationships (APA, 2022). Clinical improvements in these relationships from the present study demonstrate families’ clear ability to develop and improve this important connection (Thompson & McFerran, 2013). Second, autism literature has previously portrayed parents of youth with autism as feeling burdened and as having poor parent-child relationships (Hock & Ahmedani, 2012; Picardi et al., 2018); yet, due to PCIT’s focus on relationship-building, our results demonstrate that these positive effects are not limited to children without ASD/DD. Furthermore, older literature on attachment has previously concluded that children with autism form secure attachments less frequently than neurotypical youth, with implications for the presence and quality of the parent-child relationship (Rogers et al., 1991; Shapiro et al., 1987). While more modern views are changing these discussions (van IJzendoorn et al., 2007), this continued exploration of the parent-child relationship is critical to unpacking *how* parents and their children with ASD or other DDs relate rather than *if* they relate (Beurkens et al., 2012).

In addition to similarities in treatment involvement, children with ASD/DD in the present study demonstrated significant improvements in disruptive behavior (as characterized by reductions in Problem and Intensity scores on the ECBI) which mirrored the drops seen in the comparison sample. Of note, the current study’s sample of ASD/DD youth (on average) did not get to the targeted Intensity scores sought to graduate from PCIT (i.e., 114 [1.5 *SD* below the clinical cutoff of 131]) but the comparison sample did. The established ECBI clinical cutoff scores (131 for the Intensity Scale, 15 for the Problem Scale; Colvin et al., 1999) were derived from studies of typically developing youth ages 2–16 years. Research by Jeter and colleagues (2017) found that among families completing the ECBI, parents of children with ASD reported a higher rate of disruptive behaviors and more frequently perceived these behaviors as problematic compared to parents of typically developing youth. While several past case studies of PCIT efficacy among children with ASD have observed ECBI score reductions to nonclinical (i.e., 114) levels following treatment (e.g., Armstrong et al., 2015; Masse et al., 2016), this result has not been replicated in studies with larger sample sizes (e.g., Ginn et al., 2017; Solomon et al., 2008). However, the present study yielded no differences in Intensity scores between the two groups even with the large sample. This lack of difference in post-scores between the two groups points to improvements of the ASD/DD group still falling to near-normative levels. Moreover, given the previous discussions of restandardization of the ECBI for ASD populations (Jeter et al., 2017), these outcomes are even more impressive within this community-based setting.

These findings should be considered in light of the present study’s limitations. Firstly, this study was conducted with secondary data meaning study researchers did not manipulate which treatment was provided or when, how clinicians were trained, if alternative treatments would be more effective, or what other services youth with ASD were receiving within the state of Oregon. Therefore, we are unable to state the effectiveness of PCIT as compared to other treatment modalities for youth with ASD. However, for families receiving PCIT in the state of Oregon, we are able to say youth with ASD/DD appear to have similar outcomes on the measured variables as compared to their peers without ASD/DD.

Another limitation of the present study was that families were excluded from analyses if they had missing data. It is possible that participants with missing data or those who did not attend at least four sessions represent a meaningful subset of families participating in PCIT. Our sample also had only a small number of girls with ASD/DD. While child sex has not been found to significantly relate to treatment outcomes in the general PCIT literature, girls tend to pursue treatment for disruptive behaviors at lower rates (Bussing et al., 2003). PCIT treatment efficacy among young girls with ASD should be emphasized in future research.

It is important to note that just under 20% of families in our sample graduated from treatment, with over 80% failing to meet graduation criteria (see Table 3). This proportion was comparable for the comparison and ASD/DD groups. Community-based PCIT research has consistently reported high rates of treatment attrition (e.g., Lyon & Budd, 2010), with many families engaging in PCIT only receiving part of the treatment. Although substantial research demonstrates PCIT’s positive outcomes for families who complete it, more recent studies have revealed significant improvements with medium-to-large effect sizes even in families who terminate treatment early (Lieneman et al., 2019; Stokes et al., 2018). Past research has also implicated increased parental stress and treatment barriers (Fernandez & Eyberg, 2009) as rationale for the high attrition rates in community-implemented PCIT. Although parents of youth with ASD experience higher rates of stress and increased barriers to treatment compared to caregivers of neurotypical children (Chiri & Warfield, 2012; Hayes & Watson, 2013), we observed no significant differences in graduation rates between these groups. Indeed, factors affecting treatment completion were likely present among families completing PCIT; however, these results are promising as they indicate families of youth with ASD and/or DD in the community can graduate and benefit from PCIT at similar rates to their neurotypical peers. Nonetheless, future efforts should investigate unique treatment barriers and perspectives among families of children with ASD/DD receiving PCIT.

We also recognize that outcomes from this sample cannot be solely attributed to differences between autism and non-ASD populations. Due to uncertainty of diagnostic procedures, we decided to include all children diagnosed with a DD to be included in the ASD group in case the system of identification was unintentionally excluding children with ASD and classifying them as a more conservative DD diagnosis. Alternative explanations for DD diagnoses of these children include that it served as a catch-all for ASD-related behaviors because families did not have a proper evaluation conducted in a specialty clinic; providers knew the child was not neurotypical but did not have the expertise to narrow down a specific neurodevelopmental diagnosis; the diagnosis was provided by a primary care provider to mark a concern but did not necessarily serve as a proper diagnosis; or the diagnosis indicated concerns without additional follow-through, given that PCIT providers were not allowed to bill for these diagnostic codes. By doing so, conclusions from this study cannot be fully generalized to ASD populations. Additionally, it is possible that our current diagnostic data underestimate the actual number of children with ASD/DD in our sample. For medical insurance and reimbursement, PCIT is classified as an evidence-based treatment for disruptive behaviors, and noncompliance and disruptive behaviors are the primary conditions eligible for coverage. In Oregon, PCIT is not classified as an approved treatment for youth with ASD (Oregon Health Authority, 2020). Thus, community providers may not bill for PCIT services if a child only has a diagnosis of ASD or another neurodevelopmental condition, despite high rates of comorbid disruptive behavior and PCIT’s evidence of effectiveness with youth with ASD. Indeed, the current study contributes to the growing body of work demonstrating PCIT’s promising outcomes for children with ASD, particularly at the community level. Based on this evidence, it is recommended that governing health agencies should inform their billing policies to reflect PCIT’s benefit for youth with ASD and disruptive behavior. It is also advised that future researchers studying the dissemination of PCIT for youth with ASD form strong partnerships with community providers and oversee data reporting procedures to enhance study fidelity.

Additionally, all outcome measures in this study were based on therapist and caregiver reports. Therapist report of relationship enhancement may be biased towards expected positive change in the parent-child relationship following treatment, and caregiver reports of disruptive behavior improvement may be inflated due to the investment of time and effort in therapy (Arkes & Blumer, 1985). It is also possible that outcomes from PCIT are meaningfully impacted by agency or clinician-level variables. Extensive literature in the field of dissemination and implementation science exists (e.g., Exploration, Preparation, Implementation, Sustainment [EPIS] framework) which recognizes how inner context factors (e.g., leadership, organizational characteristics, individual clinician characteristics) may impact the successful implementation of evidence-based treatments such as PCIT (Aarons et al., 2011). However, little work has been done to recognize how these factors impact treatment implementation for youth with ASD. Future research efforts should include multiple informant outcome measures and standardized behavioral measures as well as agency- or clinician-level variables to better assess PCIT outcomes for children with ASD/DD.

Future directions in this field should investigate a wide range of treatment outcomes important for families of youth with ASD and/or DD in the community and include follow-up research for maintenance of treatment effects. Several studies have provided preliminary evidence for PCIT’s promise in improving not only child disruptive behavior, but also language production, adaptive skills, and prosocial behavior among youth with ASD (e.g., Ginn et al., 2015; Hansen & Shillingsburg, 2016; Zlomke et al., 2017). Although few studies have utilized follow-up measures to explore treatment outcomes among youth with ASD or DD following PCIT, the preliminary evidence is promising. In their study of 30 mother-child dyads, Ginn and colleagues (2015) found that improvements in child disruptive behavior and social awareness were maintained after 6 weeks following PCIT. Bagner and colleagues (2010) reported statistically significant decreases in child disruptive behavior and increases in child compliance for 28 children at risk for a DD that maintained 4 months post-PCIT. Several smaller case studies have reported maintained improvements in child behavior at 3- and 5-month follow-ups (e.g., Armstrong & Kimonis, 2013; 2015; Masse et al., 2016). Research utilizing a range of relevant outcome and follow-up measures is crucial in assessing the maintenance of treatment gains over time. This work is of particular importance in the exploration of PCIT delivered for youth with ASD and/or DD at the community level.

Further, ASD is widely regarded as a heterogenous condition and patterns of cognitive, behavioral, and social functioning may manifest differently across children (APA, 2013). Youth with ASD often have comorbid intellectual disability/global DD and language delays (Matson & Shoemaker, 2009; Munson et al., 2008). In the past, researchers have noted that PCIT may not be suitable for youth with ASD with limited receptive language capability, given the emphasis on parent-child communication (Masse et al., 2007). However, small-scale studies have begun to demonstrate the benefits of PCIT among youth with ASD and comorbid DD, reporting that positive outcomes include increased child vocalizations (Hansen & Shillingsburg, 2016). As the youth in our sample were identified by clinicians as having ASD and/or DD, it is possible that children with limited receptive language skills displayed significant improvements in disruptive behavior following PCIT. However, without adequate assessment of language skills, intellectual functioning, and/or ASD symptom severity, little is known about the clinical characteristics of the children in this group. Given the diversity of clinical presentations and child characteristics in youth with ASD, research of within-group differences for children receiving PCIT is needed.

## Conclusion

Results from the present study demonstrate that PCIT can be delivered effectively for children with ASD in community-based clinics by non-specialized clinicians. While many PCIT studies for children with ASD have explored these families’ outcomes, few have directly compared ASD/DD samples to youth without ASD/DD. These results suggest that children with ASD/DD had comparable demographics as well as similar rates in length of treatment, number of sessions, graduation, and improvement in disruptive behaviors as compared to their peers without ASD/DD. Research has consistently indicated that children with ASD have high rates of unmet behavioral health needs (Kogan et al., 2008), which is likely linked to the vast shortage of specialty clinicians trained to treat youth with ASD (Zhang & Cummings, 2020). These findings align with the growing body of literature indicating that PCIT is an effective treatment for disruptive behavior in children with ASD (e.g., Agazzi et al., 2013; Armstrong et al., 2015; Ginn et al., 2017). Moreover, these results indicate that community clinicians without formal ASD training are implementing PCIT effectively for children in this population; this is a promising step towards increasing the dissemination of evidence-based treatments for children with ASD.
